# Supplementary material for: Reduction of specific enterocytes from loss of intestinal LGR4 improves lipid metabolism in mice
Source: Nat Commun. 2024 May 23;15:4393. doi: 10.1038/s41467-024-48622-5 (PMC11116434; doi:10.1038/s41467-024-48622-5)
Supplement: Supplementary file 1 — Supplementary Information [file 41467_2024_48622_MOESM1_ESM.pdf]

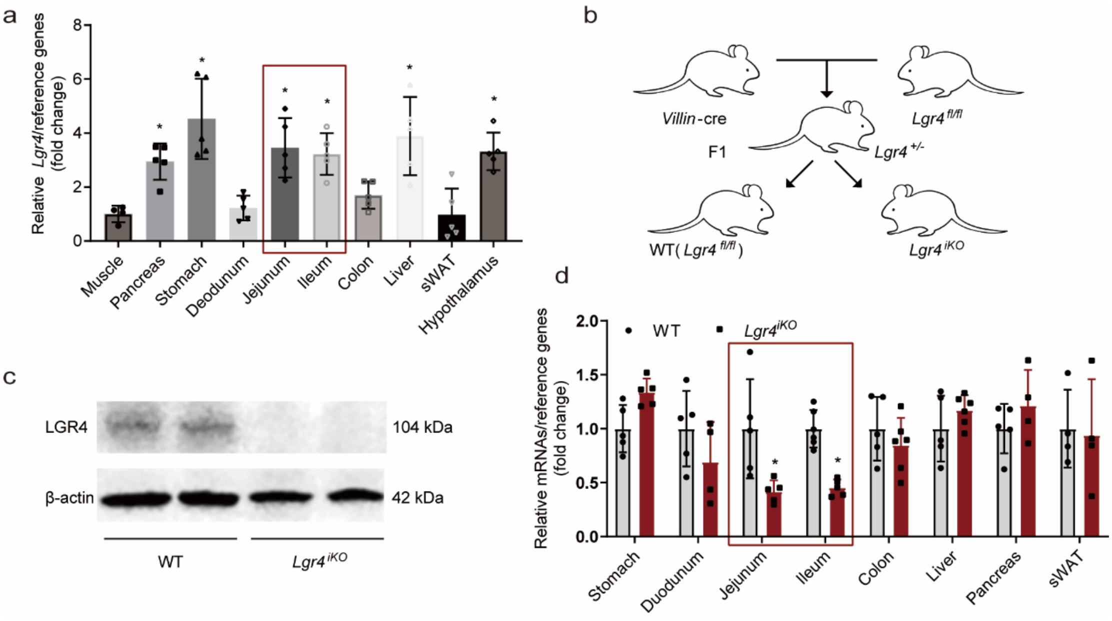

Supplementary Figure 1. Establishment of *Lgr4^{iKO}* mice

(a) *Lgr4* mRNA expression was quantified by RT-qPCR in different tissues of C57BL/6J mice and normalized to the geometric mean value of reference genes (*Hprt*, *Rpl32* and *Tbp*). Results were expressed as mean±SD. *P <0.05 vs Muscle. n=4-5.

(b) Scheme of the construct used to generate *Lgr4^{iKO}* mice and littermates. *Villin*-Cre mice were bred with *Lgr4$^{flox/flox}$* mice. The resulting F1 progeny were backcrossed to generate *Lgr4^{iKO}* mice and littermates.

(c, d) The knockout efficiency was verified by Western blot and RT-qPCR analyzing the expression levels of LGR4.

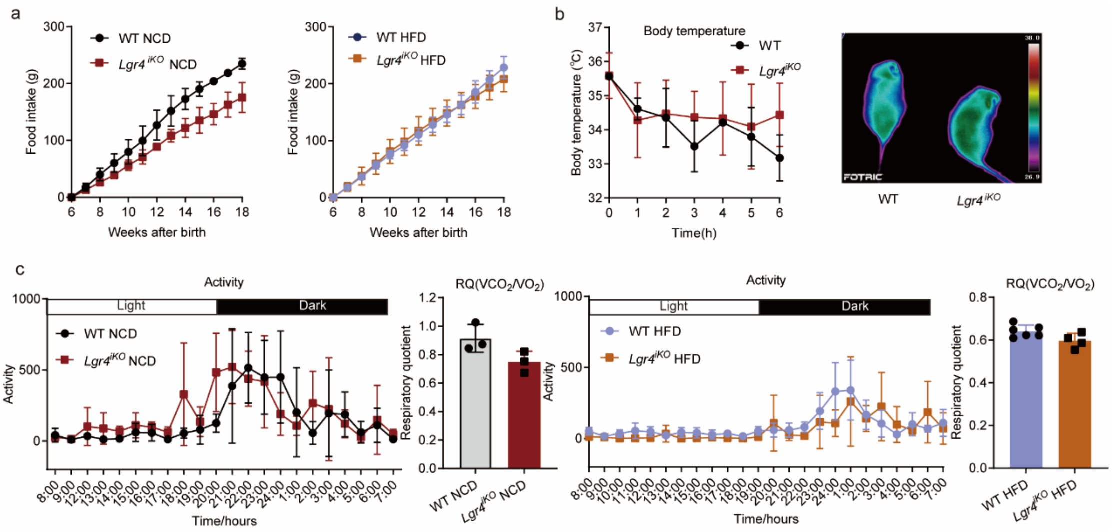

Supplementary Figure 2. Effects of intestinal *Lgr4* deletion on food intake and energy expenditure

Six-week-old male $Lgr4^{iKO}$ mice and littermates were fed normal chow diet or 60% high fat diet for 12 weeks. Results were expressed as mean±SD. n=3-6.

(a) Food intake in mice fed NCD or HFD-fed.

(b) Rectal body temperature during the cold exposure at 4°C for 6h.

(c) Locomotor activity and respiratory quotient in mice fed NCD or HFD.

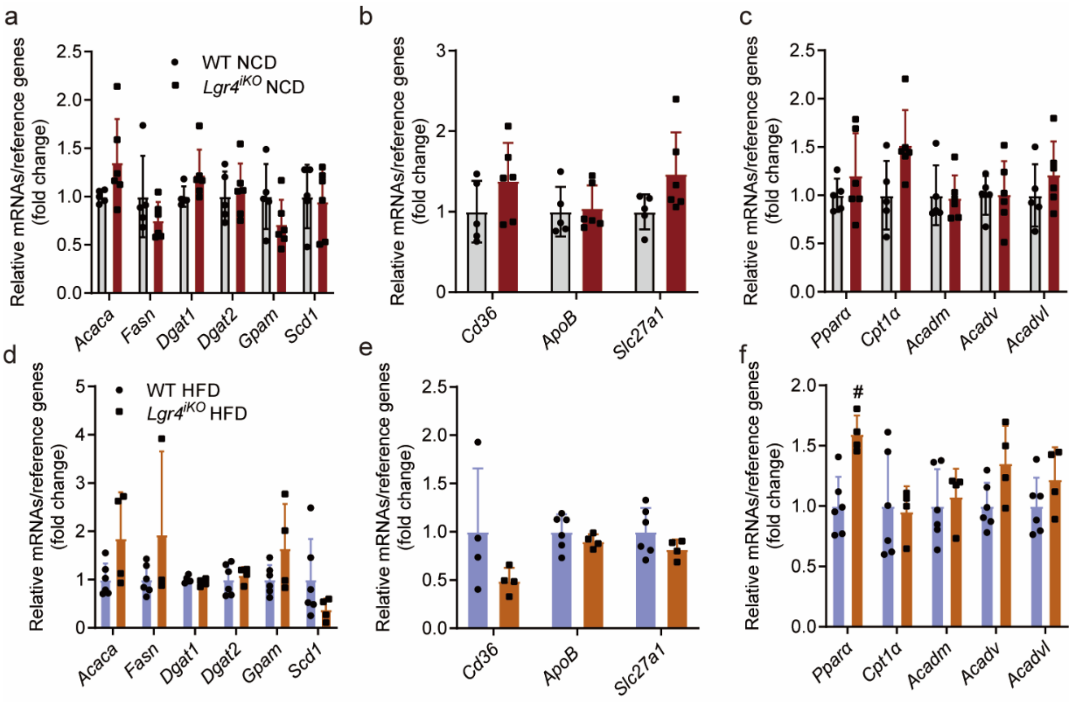

Supplementary Figure 3. Effects of intestinal *Lgr4* deletion on the mRNA levels of hepatic genes relevant to lipid metabolism

Six-week-old male *Lgr4*^{iKO} mice and littermates were fed normal chow diet or 60% high fat diet for 12 weeks. Results were expressed as mean±SD. *P<0.05 vs WT NCD. $^{\#}$P <0.05 vs WT HFD. n=3-6.

(a-c) Relative mRNA levels of hepatic genes relevant to lipogenesis, lipid transport, and β-oxidation in mice fed NCD.

(d-f) Relative mRNA levels of hepatic genes relevant to lipogenesis, lipid transport, and β-oxidation in mice fed HFD.

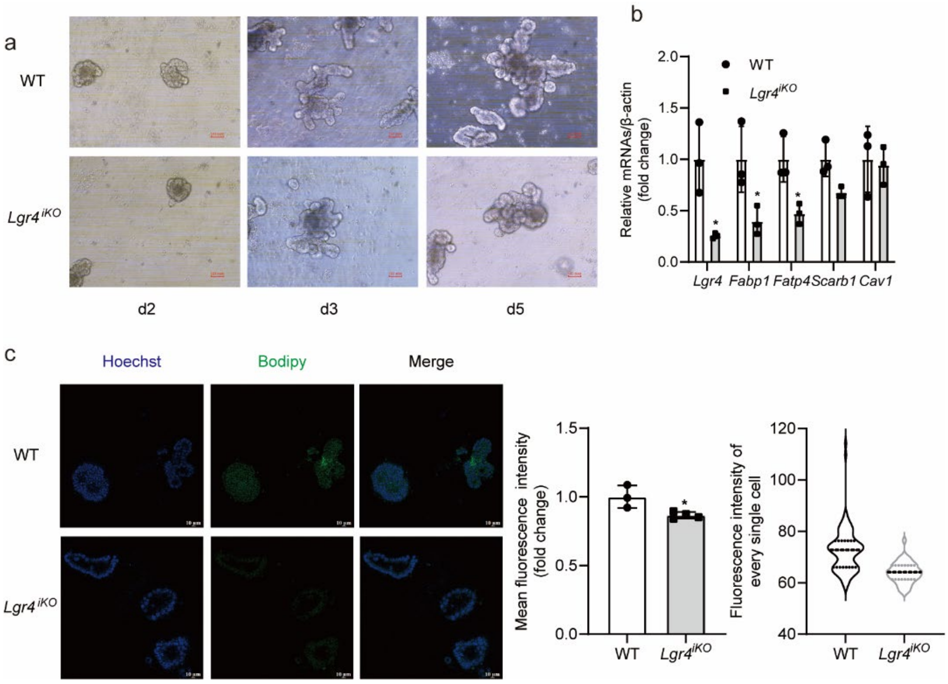

Supplementary Figure 4. Deficiency of *Lgr4* decreases lipid absorption in intestinal organoids

Organoids derived from WT and *Lgr4* deficient mice were cultured and analyzed. Results were expressed as mean±SD. *P<0.05 vs WT. n=3-4.

(a) Morphology of growth process in intestinal organoids.

(b) mRNA levels in intestinal organoids.

(c) The uptake of BODIPY-C_{12} long-chain fatty acid in intestinal organoids and the mean fluorescence intensity of each organoid and each single cell.

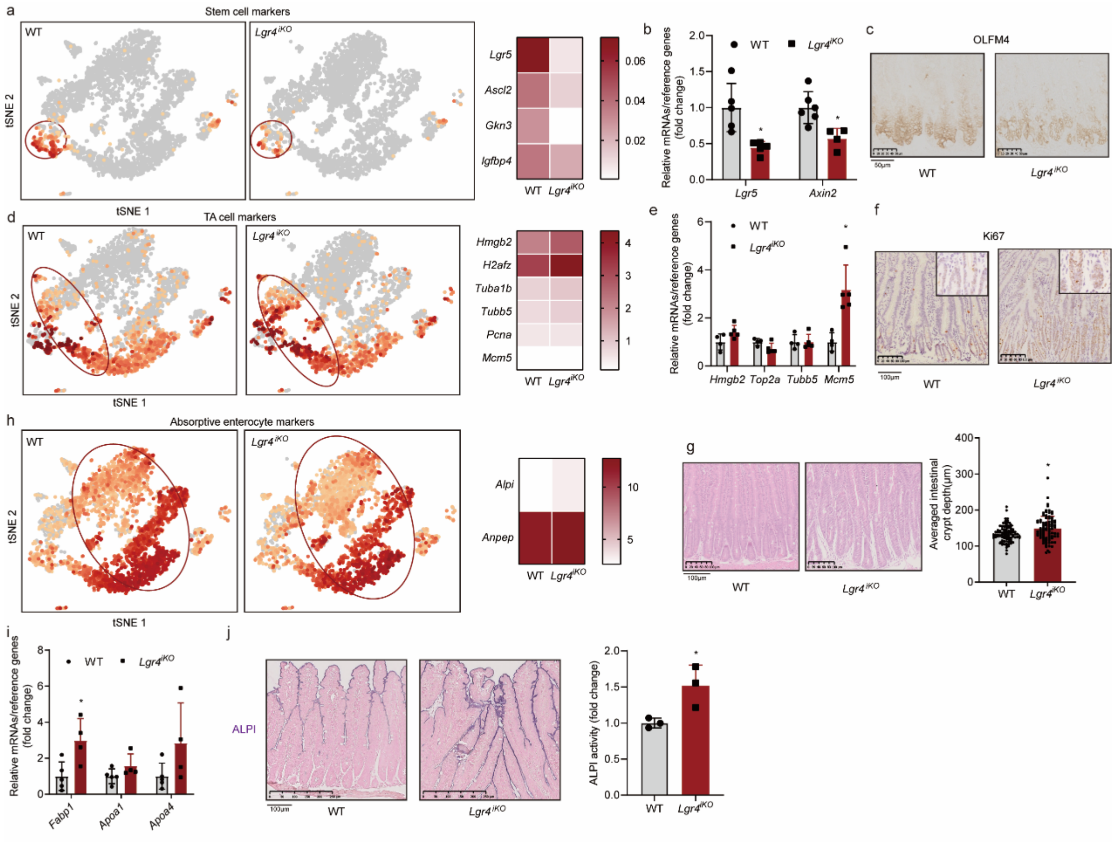

Supplementary Figure 5. Effects of intestinal *Lgr4* deletion on the number of stem cells, TA cells and absorptive enterocytes

Six-week-old male *Lgr4*^{iKO} mice and littermates were fed normal chow diet for 12 weeks. Single cell RNA sequencing was used to obtain intestinal epithelium single cell transcriptome data from 18-week-old *Lgr4*^{iKO} mice and littermates. n=3. Results were expressed as mean±SD. *P<0.05 vs WT.

(a) t-SNE plot showing stem cell marker genes expression (left) and heatmap showing UMI value (right).

(b) mRNA levels of stem cell marker genes in intestine of NCD-fed mice. n=4-6.

(c) Immunohistochemical staining of OLFM4 for stem cells in intestine.

(d) t-SNE plot showing TA cell marker genes expression (left) and heatmap showing UMI value (right).

(e) mRNA levels of TA cell marker genes in intestine of NCD-fed mice. n=4-5.

(f) Immunohistochemical staining of Ki67 for TA cells in intestine.

(g) H&E staining of the intestine and quantitative results of crypt depth.

(h) t-SNE plot showing absorptive enterocyte marker genes expression and heatmap showing UMI value.

(i) mRNA levels of absorptive enterocyte markers in intestine of NCD-fed mice. n=4-5.

(j) Detection of intestinal ALPI (an absorptive enterocyte specific marker) and quantification.

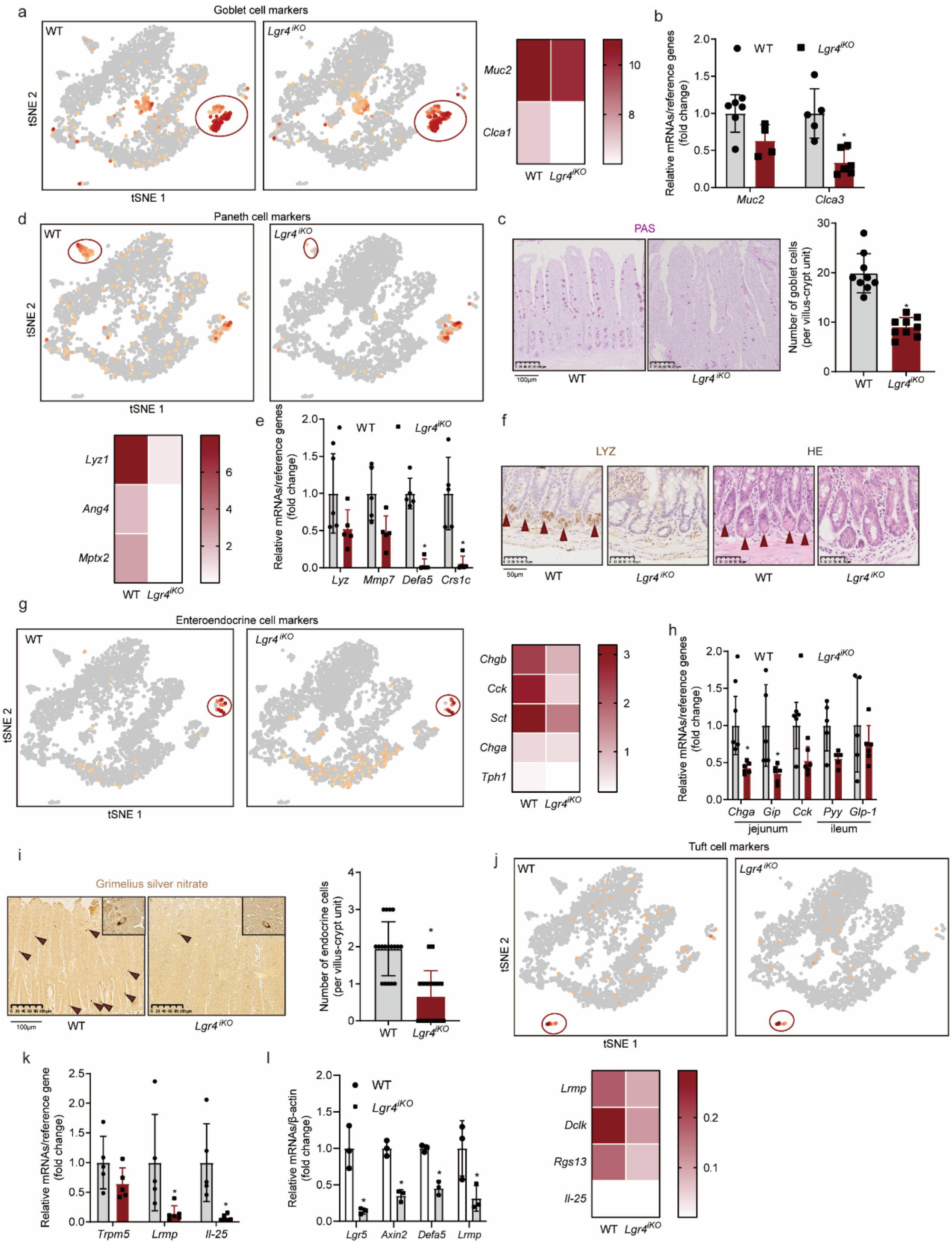

Supplementary Figure 6. Effects of intestinal *Lgr4* deletion on the number of goblet cells, Paneth cells, enteroendocrine cells and tuft cells

Six-week-old male *Lgr4*^{iKO} mice and littermates were fed normal chow diet for 12 weeks. Single cell RNA sequencing was used to obtain intestinal epithelium single cell transcriptome data from 18-week-old *Lgr4*^{iKO} mice and littermates. n=3. Results were expressed as mean±SD. *P<0.05 vs WT.

(a) t-SNE plot showing goblet cell marker genes expression and heatmap showing

UMI value.

(b) mRNA levels of goblet cell markers in intestine of NCD-fed mice. n=4-6.

(c) Detection of intestinal goblet cells by PAS staining and the number of goblet cells per villus-crypt unit.

(d) t-SNE plot showing paneth cell marker genes expression and heatmap showing UMI value.

(e) mRNA levels of paneth cell markers in intestine of NCD-fed mice. n=5.

(f) Detection of intestinal paneth cells by Immunohistochemical staining for LYZ and H&E staining.

(g) t-SNE plot showing enteroendocrine cell marker genes expression and heatmap showing UMI value.

(h) mRNA levels of enteroendocrine cell markers in intestine of NCD-fed mice. n=5-6.

(i) Intestinal enteroendocrine cells stained by Grimelius's silver nitrate method and the number of enteroendocrine cells per villus-crypt unit.

(j) t-SNE plot showing tuft cell marker genes expression and heatmap showing UMI value.

(k) mRNA levels of tuft cell markers in intestine of NCD-fed mice. n=5-6.

(l) mRNA levels of stem cell, Paneth cell and tuft cell markers of organoids derived from WT and *Lgr4* deficient mice. n=3.

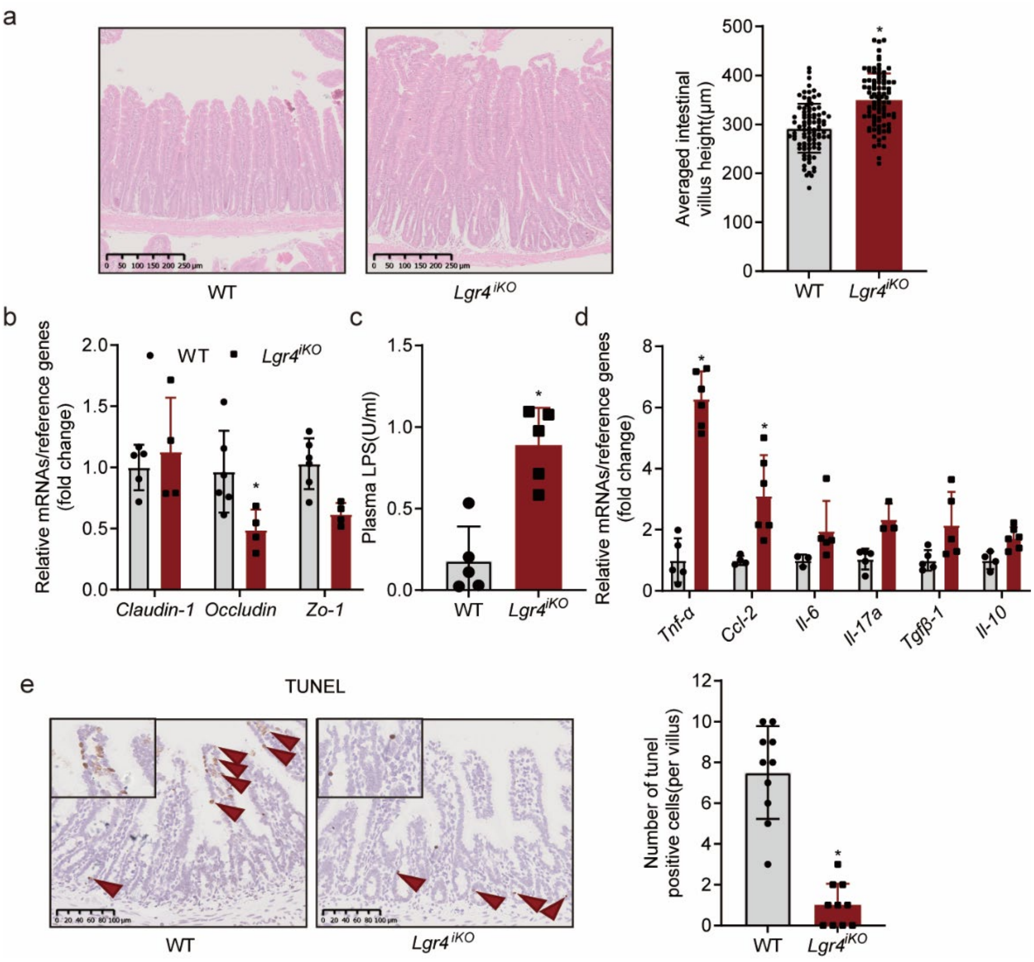

Supplementary Figure 7. Deficiency of intestinal Lgr4 disrupts the intestinal epithelial barrier and decreases villus apoptosis

Six-week-old male *Lgr4^{iKO}* mice and littermates were fed normal chow diet for 12 weeks. Results were expressed as mean±SD and analyzed by the t-test. *P<0.05 vs WT. n=3-6.

(a) H&E staining of the intestine and quantitative results of villus height.

(b) mRNA levels of tight junction related genes (*Claudin-1, Occludin and Zo-1*).

(c) Plasma levels of LPS (Lipopolysaccharides).

(d) mRNA levels of inflammation-related genes (*Tnf-α, Ccl-2, Il-6, Il-17a, Tgf-β1 and Il-10*). These genes were determined by real-time quantitative PCR and normalized to the geometric mean value of reference genes (*Hprt, Rpl32 and Tbp*).

(e) TUNEL staining labeled apoptotic cells and the number of apoptotic cells per villus.

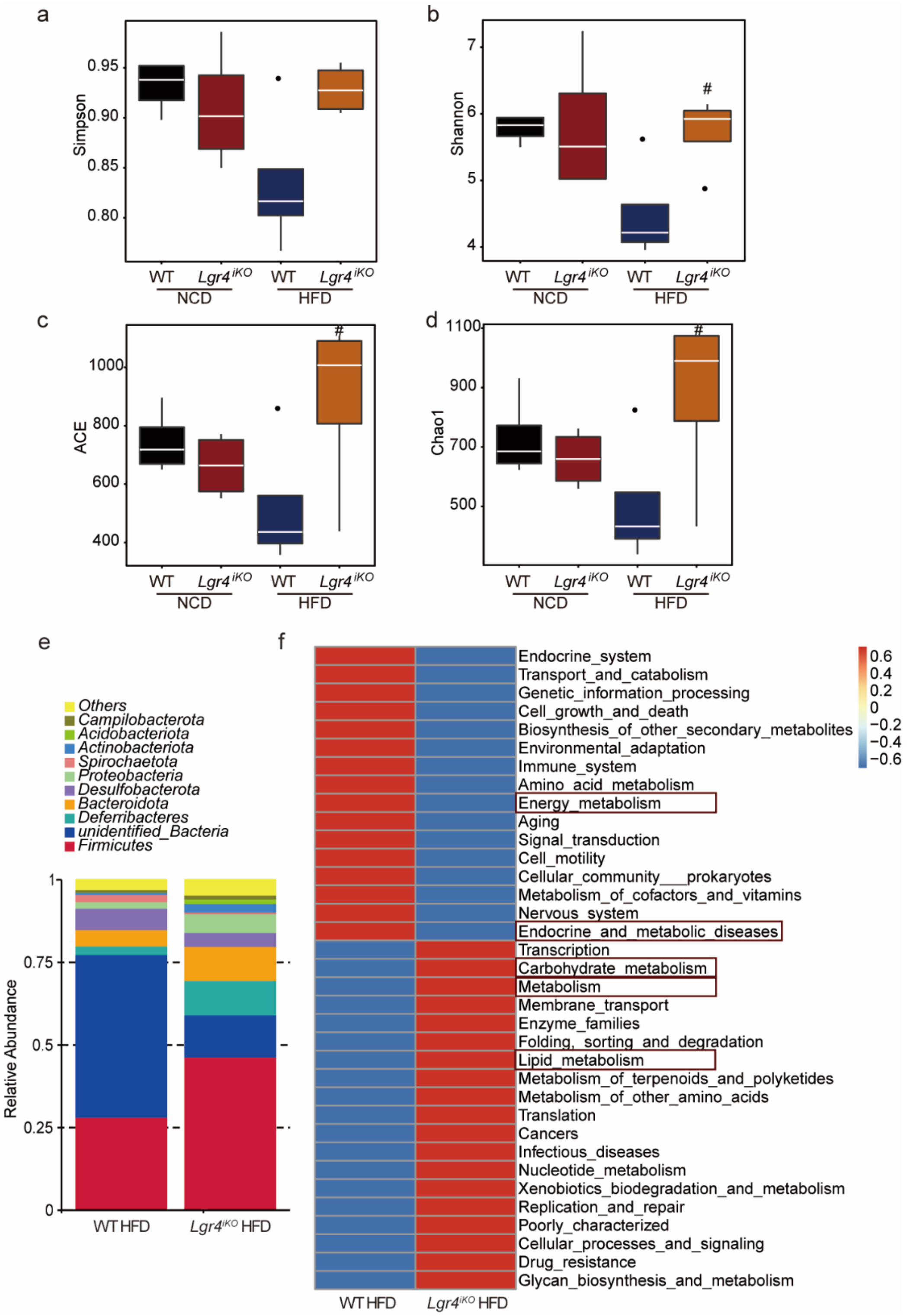

Supplementary Figure 8. Deficiency of intestinal *Lgr4* disrupts the microbiota homeostasis

Six-week-old male *Lgr4*^{iKO} mice and littermates were fed normal chow diet or 60% high fat diet for 12 weeks. #P<0.05 vs WT HFD. n=4.

(a-d) Alpha-diversity-Simpson, shannon, ACE and chao1 diversity index of WT and Lgr4iKO mice, pairwise significance determined by Wilcox test.

(e) Phylum level bacterial relative abundance.

(f) The relative abundance cluster analysis of the functional pathway shows the top 35 functional pathways.

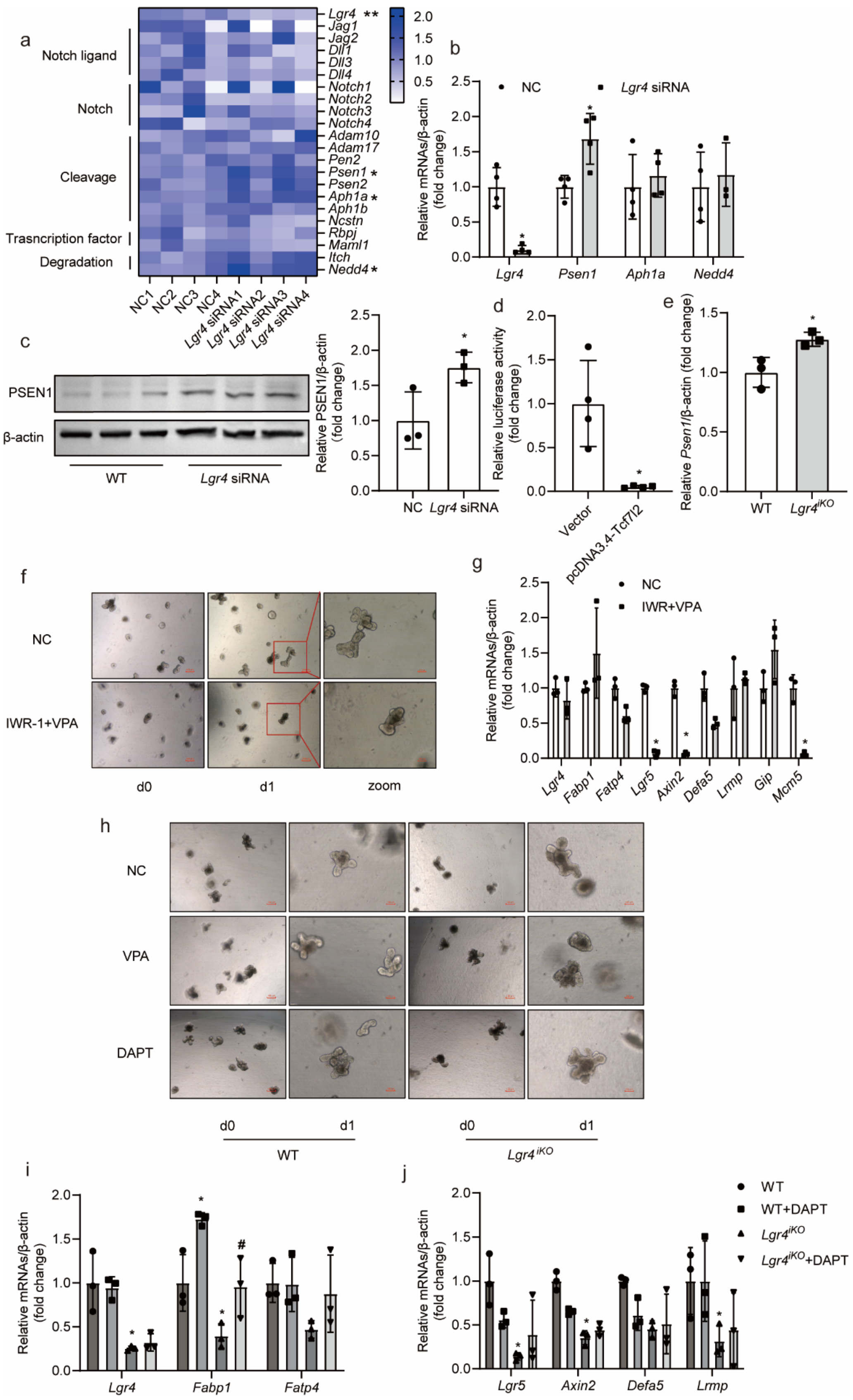
a
Notch ligand
Notch
Cleavage
Trasncription factor
Degradation
Lgr4 **
Jag1
Jag2
Dll1
Dll3
Dll4
Notch1
Notch2
Notch3
Notch4
Adam10
Adam17
Pen2
Psen1 *
Psen2
Aph1a *
Aph1b
Ncstn
Rbpj
Maml1
Itch
Nedd4 *
NC1 NC2 NC3 NC4 Lgr4 siRNA1 Lgr4 siRNA2 Lgr4 siRNA3 Lgr4 siRNA4
b
NC
Lgr4 siRNA
Relative mRNAs/β-actin (fold change)
Lgr4
Psen1
Aph1a
Nedd4
c
PSEN1
β-actin
WT
Lgr4 siRNA
Relative PSEN1/β-actin (fold change)
NC
Lgr4 siRNA
d
Relative luciferase activity (fold change)
Vector
pcDNA3.4-Tcf7l2
e
Relative Psen1/β-actin (fold change)
WT
Lgr4iKO
f
NC
IWR-1+VPA
d0
d1
zoom
g
NC
IWR+VPA
Relative mRNAs/β-actin (fold change)
Lgr4
Fabp1
Fatp4
Lgr5
Axin2
Defa5
Lrmp
Gip
Mcm5
h
NC
VPA
DAPT
d0
d1
WT
d0
d1
Lgr4iKO
i
Relative mRNAs/β-actin (fold change)
Lgr4
Fabp1
Fatp4
j
Relative mRNAs/β-actin (fold change)
Lgr5
Axin2
Defa5
Lrmp
WT
WT+DAPT
Lgr4iKO
Lgr4iKO+DAPT

Supplementary Figure 9. The relationship between LGR4 and Notch signaling

MODE-K, IEC6 cell lines and organoids were used to explore the relationship of LGR4 and Notch signaling. Results were expressed as mean±SD. *P<0.05 vs WT.

(a-c) The mouse small intestinal epithelial cell line MODE-K cells and IEC6 cells were transfected with *Lgr4* siRNA for 48 hours. n=3-4. (a) mRNA levels of genes of Notch signaling in MODE-K cells. (b) mRNA levels in IEC6 cells. (c) PSEN1 protein level in IEC6 cells.

(d) pGL3-Psen1 promoter, pRL-TK and pcDNA3.4-Tcf7l2 or empty vector plasmids were co-transfected into 293T cells for 24h. The relative luciferase activity. n=4.

(e) *Psen1* mRNA level in organoids derived from WT and *Lgr4* deficient mice, n=3.

(f, g) Organoids derived from WT mice were incubated with 10 μM IWR-1 and 4 mM VPA or DMSO as a negative control for 1 day, n=3. (f) Representative figures. (g) mRNA levels of genes of lipid absorption and differentiation.

(h-j) Organoids derived from WT and *Lgr4* deficient mice were incubated with 40 μM DAPT or DMSO as a negative control for 1 day, n=3. (h) Representative figures. (i) mRNA levels of genes related to lipid absorption. (j) mRNA levels of genes related to cell differentiation. $^{\#}$P<0.05 vs $Lgr4^{iKO}$.

Supplementary Table 1

Primers for Quantitative RT-PCR

Genes	Upstream primer (5'-3')	Downstream primer (5'-3')
Lgr4	CCATTCGTGGACTGAGTGCT	GTTGGTGAATGCGAAGTCGG
Ucp1	GGACGACCCCTAATCTAATG	CATTAGATTAGGGGTCGTCC
Ucp3	ATCAGGATTCTGGCAGGC	GCCTGCCAGAATCCTGAT
Pgc1α	GATTGAAGTGGTGTAGCGAC	GTCGCTACACCACTTCAATC
Acaca	TGGTCGTGACTGCTCTGTGC	GTAGCCGAGGGTTCAGTTCC
Fasn	TGGGTTCTAGCCAGCAGAGT	ACCACCAGAGACCGTTATGC
Dgat1	TTCCGCCTCTGGGCATT	AGAATCGGCCCACAATCCA
Dgat2	CGTGACGTGCATTGGCTTC	TGGAGGGCTGAGAGGATGC
Gpam	CACACGAGCAGGAAAGATGA	GGACTGCATAGATGCTGCAA
Scd1	GCGATACACTCTGGTGCTCA	CCCAGGGAAACCAGGATATT
Cd36	TGGTCAAGCCAGCTAGAAA	CCCAGTCTCATTTAGCCAC
ApoB	TCACCATTTGCCCTCAACCTAA	GAAGGCTCTTTGGAAGTGTAAAC
Slc27a1	CAGTGCCACCAACAAGAAGA	CAGCTCGTCCATCACTAGCA
Pparα	GAGAAGTTGCAGGAGGGGATTGTG	AAGACTACCTGCTACCGAAATGGG
Cpt1α	ATCGTGGTGGTGGGTGTGATAT	ACGCCACTCACGATGTTCTTC
Acadm	TTACCGAAGAGTTGGCGTATGG	TGCGGAGGGCTCTGTCAC
Acadv	CTCCCTGCGCGTCCTGAG	AAAATGTCATGCTCCGAGGAAAAG
Acadvl	GCCCAGACACACAACCTTTG	CCGAGCCGACTGCATCTC
Fatp4	ACTGTTCTCCAAGCTAGTGCT	GATGAAGACCCGGATGAAACG
Fabp2	GTGGAAAGTAGACCGGAACGA	CCATCCTGTGTGATTGTCAGTT
Cav1	GCGACCCCAAGCATCTCAA	ATGCCGTCGAAACTGTGTGT
Glut1	TCAAACATGGAACCACCGCTA	AAGAGGCCGACAGAGAAGGAA
Glut2	TTCCAGTTCGGCTATGACATCG	CTGGTGTGACTGTAAGTGGGG
Glut5	TCTCTTCCAACGTGGTCCCTA	GAGACTCCGAAGGCCAAACAG
Sglt1	CACCGAGGGCTGACTCATTC	TGATCCGTACACCAGTACCAC
Lgr5	GCTGGAGAGGAACGACATCC	CAGCGGCCCTTGTGTAAGTA
Axin2	AACCTATGCCCGTTTCCTCTA	GAGTGTAAAGACTTGGTCCACC
Hmgb2	AAGAGCGACAAAGCTCGTTATG	GCAGTATCTCCAATAGACAGGC
Top2a	AACAAAGGGACCCAAAAATGTCT	TGTGTTCAACAACAGGGATTCC
Tubb5	CAACTTCGTTTTCGGTCAGTCT	GACAGAGTCAACCAACTCAGC

Mcm5	CCTGAGACAGTACCGAGTGG	GTAATGCCGCTTGAGTTCATCT
Fabp1	GTCAGAAATCGTGCATGAAGGG	GAACTCATTGCGGACCACTTT
Apoa1	GCTCAAGAGCAACCCTACCTT	GCTTTCTCGCCAAGTGTCTTC
Apoa4	CCAATGTGGTGTGGGATTACTT	AGTGACATCCGTCTTCTGAAAC
Muc2	AGGGCTCGGAACTCCAGAAA	CCAGGGAATCGGTAGACATCG
Clca3	AAGCCTGAATATACGAGGCCA	GGGCTCATCATTGCCTAGAGG
Lyz	GAGACCGAAGCACCGACTATG	CGGTTTTGACATTGTGTTCGC
Mmp7	CTTACCTCGGATCGTAGTGGA	CCCCAACTAACCCTCTTGAAGT
Defa5	CAGGCTGATCCTATCCACAA	CAGAGCCGATGGTTGTCATA
Crs1c	TCCCAGCCATGAAGACACTA	GCACCACGGAAACCTTCTTT
Chga	CCAAGGTGATGAAGTGCGTC	GGTGTCGCAGGATAGAGAGGA
Gip	TGAGTTCCGATCCCATGCTAA	GGCGATGCTGTAATCACTGATG
Cck	TGGATGGTGACCTCTGGTTG	GCAGGAAAAATTGGGTTCGTGAG
Pyy	ACGGTCGCAATGCTGCTAAT	GACATCTCTTTTTCCATACCGCT
Glp-1	TTACTTTGTGGCTGGATTGCTT	AGTGGCGTTTGTCTTCATTCA
Trpm5	CCAGCATAAGCGACAACATCT	GAGCATACAGTAGTTGGCCTG
Lrmp	ATGGAGACTACACGAGTGTGG	CTGCCCTTCCCTCCGTTTT
Il-25	ACAGGGACTTGAATCGGGTC	TGGTAAAGTGGGACGGAGTTG
Ccnb1	CTTGCAGTGAGTGACGTAGAC	CCAGTTGTCGGAGATAAGCATAG
Cdc20	CAGCCTGGAGACTACATATCCT	CGGAGTGACTGGTCATGTTTC
Cenpa	GCCGTGGTGTGGATTTTTGG	GAGGTAGGCGTCCTCAAAGAG
Cdkn3	ACCCTGATACATTGTTACGGAGG	CTCGAAGGCTGTCTATGGCTT
Ube2c	GAGTCAGACAACCTGTTCAAGTG	TCTAGGGAGAGTTTGTACCTCAG
Dll1	GCAGGACCTTCTTTCGCGTAT	AAGGGGAATCGGATGGGGTT
Math1	GGGGTTGTAGTGGACGAGC	CGTTGTTGAAGGACGGGATAAC
Hes1	CCAGCCAGTGTCAACACGA	AATGCCGGGAGCTATCTTTCT
Ccnd1	GCGTACCCTGACACCAATCTC	ACTTGAAGTAAGATACGGAGGGC
Hey1	GCGCGGACGAGAATGGAAA	TCAGGTGATCCACAGTCATCTG
Scarb1	AAACAGGGAAGATCGAGCCAG	GGTCTGACCAAGCTATCAGGTT
Claudin-1	CTGGAAGATGATGAGGTGCAGAAG	CCACTAATGTCGCCAGACCTGAA
Occludin	GCCTATGGAACGGGCATCTT	GCCAGCAGGAAACCCTTTG
Zo-1	GAGGCTTCAGAACGAGGCTATT	CATGTCGGAGAGTAGAGGTTCGA
Tnf-α	CCAGACCCTCACACTCAGATC	CACTTGGTGGTTTGCTACGAC
Ccl-2	TAAAAACCTGGATCGGAACCAAA	GCATTAGCTTCAGATTTACGGGT

Il-6	CTGCAAGAGACTTCCATCCAG	AGTGGTATAGACAGGTCTGTTGG
Il-17a	CCTCAGACTACCTCAACCG	CTCCCTCTTCAGGACCAG
Tgf-β1	CTTCAATACGTCAGACATTCGGG	GTAACGCCAGGAATTGTTGCTA
Il-10	CTTACTGACTGGCATGAGGATCA	GCAGCTCTAGGAGCATGTGG
Jag1	CCTCGGGTCAGTTTGAGCTG	CCTTGAGGCACACTTTGAAGTA
Jag2	CAATGACACCACTCCAGATGAG	GGCCAAAGAAGTCGTTGCG
Dll3	CTGGTGTCTTCGAGCTACAAAT	TGCTCCGTATAGACCGGGAC
Dll4	CAGTTGCCCTTCAATTTCACCT	AGCCTTGGATGATGATTTGGC
Notch1	CCCTTGCTCTGCCTAACGC	GGAGTCCTGGCATCGTTGG
Notch2	CTGTGAGCGGAATATCGACGA	ATAGCCTCCGTTTCGGTTGG
Notch3	AGTGCCGATCTGGTACAACTT	CACTACGGGGTTCTCACACA
Notch4	CCAGAATGCGAGACAGAACTG	CAGGTGCAGGAATAGCCCTC
Adam10	ATGGTGTTGCCGACAGTGTTA	GTTTGGCACGCTGGTGTTTTT
Adam17	ACCACTTTGGTGCCTTTCGT	GTCGCAGACTGTAGATCCCTT
Pen2	ATGAACTTGGAGCGGGTATCC	CGAGGAACGCCTCTCTGAAG
Psen1	ATACCTGCACCTTTGTCCTACT	GCTCAGGGTTGTCAAGTCTCT
Psen2	GAAGACTCCTACGACAGTTTTGG	CACCAGGACGCTGTAGAAGAT
Aph1a	TGCTGTGTTTTTCGGATGCAC	TCTGATCGGTCTGTCACATGG
Aph1b	CCGCGCTCGCTCTTTATGT	TGTACTGGTCCATCTCTGTTGT
Ncstn	CTCAACGCCACTCATCAGATT	ACTGGTTGTTCCCTTCAGCTT
Rbpj	CTCCACCCAAACGACTCACTA	TCCAACCACTGCCCATAAGATA
Maml1	CGTAGCTCAGAGCAACCTCAT	TTCATGTCTTCGTCGGGCAC
Itch	TGGGTAGTCTGACCATGAAATCT	GGGGTAACAATAACTGTGAGGG
Nedd4	TTCACTGCTGATCCGTACCTG	GGTAAGGATTCCACTCATCGGG
Rat-Actb	GCTGTGCTATGTTGCCCTAGACTTC	GGAACCGCTCATTGCCGATAGTG
Rat-Lgr4	TTCAGTTTCCATCAGCAGCCAAGG	CAGCAGTCACAGACAGTCAGGTTAC
Rat-Psen1	CCATTCACAGAAGACACCGAGAC	TTATACAGGACCACCAGGAGGATG
Rat-Psen2	GATCATGCTGTTCGTGCCTGTC	TGCCCGTTCTTCTCCGTGTAG
Rat-Nedd4	TGGAGGAGTTGCCCGAGAATG	ACAAGCCCGAGTTTGGATTTATCTG
