# Supplementary material for: Reduction of specific enterocytes from loss of intestinal LGR4 improves lipid metabolism in mice
Source: Nat Commun. 2024 May 23;15:4393. doi: 10.1038/s41467-024-48622-5 (PMC11116434; doi:10.1038/s41467-024-48622-5)
Supplement: Supplementary file 3 — Reporting Summary [file 41467_2024_48622_MOESM3_ESM.pdf]

nature portfolio

Corresponding author(s): Yue Yin and Weizhen Zhang

Last updated by author(s): Apr 18, 2024

Reporting Summary

Nature Portfolio wishes to improve the reproducibility of the work that we publish. This form provides structure for consistency and transparency in reporting. For further information on Nature Portfolio policies, see our Editorial Policies and the Editorial Policy Checklist.

Statistics

For all statistical analyses, confirm that the following items are present in the figure legend, table legend, main text, or Methods section.

n/a	Confirmed	
☐	☒	The exact sample size (*n*) for each experimental group/condition, given as a discrete number and unit of measurement
☐	☒	A statement on whether measurements were taken from distinct samples or whether the same sample was measured repeatedly
☐	☒	The statistical test(s) used AND whether they are one- or two-sided<br>*Only common tests should be described solely by name; describe more complex techniques in the Methods section.*
☐	☒	A description of all covariates tested
☐	☒	A description of any assumptions or corrections, such as tests of normality and adjustment for multiple comparisons
☐	☒	A full description of the statistical parameters including central tendency (e.g. means) or other basic estimates (e.g. regression coefficient) AND variation (e.g. standard deviation) or associated estimates of uncertainty (e.g. confidence intervals)
☐	☒	For null hypothesis testing, the test statistic (e.g. *F*, *t*, *r*) with confidence intervals, effect sizes, degrees of freedom and *P* value noted<br>*Give P values as exact values whenever suitable.*
☒	☐	For Bayesian analysis, information on the choice of priors and Markov chain Monte Carlo settings
☒	☐	For hierarchical and complex designs, identification of the appropriate level for tests and full reporting of outcomes
☒	☐	Estimates of effect sizes (e.g. Cohen's *d*, Pearson's *r*), indicating how they were calculated

Our web collection on statistics for biologists contains articles on many of the points above.

Software and code

Policy information about availability of computer code

Data collection	(1) Single cell capture, library preparation, sequencing and data analysis were performed by Capitalbio Technology Corporation (Beijing, China). Single cell capture and library preparation were performed following general protocols.<br>(2)The purified RNA was quantified using a NanoDrop spectrophotometer (Agilent Alto, CA, USA).
Data analysis	GraphPad Prism 9.5, Image J 2.9.0 , Loupe Browser 5.0

For manuscripts utilizing custom algorithms or software that are central to the research but not yet described in published literature, software must be made available to editors and reviewers. We strongly encourage code deposition in a community repository (e.g. GitHub). See the Nature Portfolio guidelines for submitting code & software for further information.

Data

Policy information about availability of data

All manuscripts must include a data availability statement. This statement should provide the following information, where applicable:

- Accession codes, unique identifiers, or web links for publicly available datasets
- A description of any restrictions on data availability
- For clinical datasets or third party data, please ensure that the statement adheres to our policy

The source data supporting the conclusions of this article will be made available by the authors, without undue reservation. Raw sequences have been deposited on NCBI public repository (Bioproject # PRJNA 981236 and PRJNA 997814).

Research involving human participants, their data, or biological material

Policy information about studies with human participants or human data. See also policy information about sex, gender (identity/presentation), and sexual orientation and race, ethnicity and racism.

Reporting on sex and gender	n/a
Reporting on race, ethnicity, or other socially relevant groupings	n/a
Population characteristics	n/a
Recruitment	n/a
Ethics oversight	n/a

Note that full information on the approval of the study protocol must also be provided in the manuscript.

Field-specific reporting

Please select the one below that is the best fit for your research. If you are not sure, read the appropriate sections before making your selection.

☒ Life sciences ☐ Behavioural & social sciences ☐ Ecological, evolutionary & environmental sciences

For a reference copy of the document with all sections, see nature.com/documents/nr-reporting-summary-flat.pdf

Life sciences study design

All studies must disclose on these points even when the disclosure is negative.

Sample size	No sample size calculation was performed. For animal experiments, we bred transgenic animals to get male Lgr4iKO and their littermate WT mice of the same or similar age (the gap of age is within two weeks). When they grown up to six to eight weeks old, these mice were fed on HFD/NCD for 12 weeks, and the sample size depended on the number of each batch of mice born. Overall, n=3-11 for NCD-fed mice, and n=4-6 for HFD-fed mice, and additional details regarding sample size are described in figure legends and can be found in Source Data. We included as many animals as possible in our experiments and ensured that at least three animals were included in each group so that statistics make sense.
Data exclusions	PCR data were excluded based on sample degradation, observed by the expression of reference genes.
Replication	For animal experiments, at least three animals were included in each group. For experiments on cell lines and organoids, the experiments were independently repeated at least for three times. For scRNA-seq analysis, single cell suspension from three animals of each group were mixed together and analyzed. Finally, we got scRNA-seq data of abundant cells from Lgr4iKO and WT littermates. Considering that the cells were heterogeneous and further verification experiments would be taken out, we think it is not necessary to perform more replications.
Randomization	For animal experiments, Lgr4iKO mice and littermates were divided randomly into NCD and HFD groups. For experiments in vitro, the cells or organoids of different groups received the same treatment, therefore, randomization was unnecessary.
Blinding	Animal treatments were not done blind. However, standard randomization procedures were strictly followed with proper controls included in all experiments. Biological samples, and physiological data were collected and analyzed under the same conditions.

Reporting for specific materials, systems and methods

We require information from authors about some types of materials, experimental systems and methods used in many studies. Here, indicate whether each material, system or method listed is relevant to your study. If you are not sure if a list item applies to your research, read the appropriate section before selecting a response.

Materials & experimental systems

n/a	Involved in the study
☐	☒ Antibodies
☐	☒ Eukaryotic cell lines
☒	☐ Palaeontology and archaeology
☐	☒ Animals and other organisms
☒	☐ Clinical data
☒	☐ Dual use research of concern
☒	☐ Plants

Methods

n/a	Involved in the study
☒	☐ ChIP-seq
☐	☒ Flow cytometry
☒	☐ MRI-based neuroimaging

Antibodies

Antibodies used

For WB:
Rabbit anti-LGR4, Abcam, ab75501, 1:1000
Rabbit anti-CD36, Proteintech, 18836-1-AP, 1:1000
Rabbit anti-LaminB1, Proteintech, 12987-1-AP, 1:2000
Mouse anti-β-actin, Proteintech, 66009-1-Ig, 1:5000
Rabbit anti-β-catenin, Cell Signaling Technology, 8480, 1:1000
Rabbit anti- PSEN1, ABclonal, A2187, 1:1000
Rabbit anti-HES1, ABclonal, A0925, 1:1000
Rabbit anti-FATP4, Abmart, T57249, 1:1000

For IHC:
Rabbit anti-Ki67, Abcam, ab15580, 1:200
Rabbit anti-OLFM4, Cell Signaling Technology, 39141, 1:200
Rabbit anti-LYZ, ABclonal, A13511, 1:200
Rabbit anti-GLUT2, ABclonal, A9843, 1:200
Rabbit anti-FATP4, Abmart, T57249, 1:200

Validation

Rabbit anti-LGR4, Abcam, ab75501
https://www.abcam.cn/products/primary-antibodies/gpcr-gpr48lgr4-antibody-ab75501.html

Rabbit anti-CD36, Proteintech, 18836-1-AP
https://www.ptgcn.com/products/CD36-Antibody-18836-1-AP.htm

Rabbit anti-LaminB1, Proteintech, 12987-1-AP
https://www.ptgcn.com/products/LMNB1-Antibody-12987-1-AP.htm

Mouse anti-β-actin, Proteintech, 66009-1-Ig
https://www.ptgcn.com/products/Pan-Actin-Antibody-66009-1-Ig.htm

Rabbit anti-β-catenin, Cell Signaling Technology, 8480
https://www.cellsignal.cn/products/primary-antibodies/b-catenin-d10a8-xp-174-rabbit-mab/8480

Rabbit anti- PSEN1, ABclonal, A2187
https://abclonal.com.cn/catalog/A2187

Rabbit anti-HES1, ABclonal, A0925
https://abclonal.com.cn/catalog/A0925

Rabbit anti-FATP4, Abmart, T57249
http://www.ab-mart.com.cn/page.aspx?node=%2077%20&id=%2049225

Rabbit anti-Ki67, Abcam, ab15580
https://www.abcam.cn/products/primary-antibodies/ki67-antibody-ab15580.html

Rabbit anti-OLFM4, Cell Signaling Technology, 39141
https://www.cellsignal.cn/products/primary-antibodies/olfm4-d6y5a-xp-rabbit-mab/39141

Rabbit anti-LYZ, ABclonal, A13511
https://abclonal.com.cn/catalog/A13511

Rabbit anti-GLUT2, ABclonal, A9843
https://abclonal.com.cn/catalog/A9843

Eukaryotic cell lines

Policy information about cell lines and Sex and Gender in Research

Cell line source(s)	MODE-K cell line was obtained from Shanghai Huzhen Industrial Co., Ltd (Shanghai, China). IEC6 cell line was generously gifted by Wang Quan lab, The First Hospital of Jilin University, and it was obtained from EK-Bioscience (Shanghai, China). 293T cell line was obtained from ATCC (USA).
Authentication	Authentication was done by manufacturers and no further authentication was performed by us before use.
Mycoplasma contamination	Cell lines were routinely monitored for mycoplasma contamination and negative results were obtained.
Commonly misidentified lines (See ICLAC register)	No commonly misidentified cell lines were used.

Animals and other research organisms

Policy information about studies involving animals; ARRIVE guidelines recommended for reporting animal research, and Sex and Gender in Research

Laboratory animals	Villin-Cre mice (gifted by Professor Jiang Changtao, Peking University) were bred with Lgr4flox/flox mice (from Helmholtz Zentrum, Germany) to generate Lgr4iKO mice. The animals were C57BL/6J background. Six to eight-week old animals were fed with NCD or HFD for 12 weeks and sacrificed for further experiments.
Wild animals	No wild animals used in this study.
Reporting on sex	Male mice of Lgr4iKO and WT controls were used in this study; while female mice were used for breeding.
Field-collected samples	No field-collected samples was used.
Ethics oversight	All experiments were conducted in strict accordance with the Guide for the Care and Use of Laboratory Animals prepared by the National Academy of Sciences (NIH publication 86-23, revised 1985). Experimental protocols were approved by the Animal Care and Use Committee of Peking University.

Note that full information on the approval of the study protocol must also be provided in the manuscript.

Flow Cytometry

Plots

Confirm that:

- [x] The axis labels state the marker and fluorochrome used (e.g. CD4-FITC).
- [x] The axis scales are clearly visible. Include numbers along axes only for bottom left plot of group (a 'group' is an analysis of identical markers).
- [x] All plots are contour plots with outliers or pseudocolor plots.
- [x] A numerical value for number of cells or percentage (with statistics) is provided.

Methodology

Sample preparation	MODE-K cells were cultured in a humid atmosphere (5% CO2) using RPMI-1640 culture medium supplemented with 10% FBS and 1% penicillin/streptomycin at 37°C. Cells were seeded in 12-well plate and grown until 70% confluency before transfection. Cells were washed with sterile phosphate-buffered saline (PBS) before transfection, then incubated with 50nM Lgr4 siRNA or non-targeting Control siRNA (Synbio Technologies, Suzhou, China) for 48 hours in a volume of 1ml/well. Cells were then treated with BODIPY-C12 long-chain fatty acid (Invitrogen, Carlsbad, CA, USA) for 2h.
Instrument	BD FACS Calibur flow cytometer
Software	FlowJo v10
Cell population abundance	100 000 events per sample were analysed.
Gating strategy	The population of a medium size is selected in forward and side scatter to exclude clumps and small debris. We used unlabeled cells as negative controls.

- [ ] Tick this box to confirm that a figure exemplifying the gating strategy is provided in the Supplementary Information.
